# An evaluation of factors associated with hypertension in children and adolescents with obesity

**DOI:** 10.3389/fcvm.2026.1805009

**Published:** 2026-05-28

**Authors:** Kexin Zhou, Zili Cai, Shiting Xiang, Jianhui Wei, Manxin Deng, Yunlong Peng, Zhiyu Liu, Jie Tan, Guanghui Zhu, Zhuo Gong, Qi Li, Lemei Zhu, Jun Qiu

**Affiliations:** 1 Pediatrics Research Institute of Hunan Province, Hunan Children’s Hospital, Changsha, China; 2 School of Public Health, Changsha Medical University, Changsha, China; 3 Child Health Development Center, Hunan Children’s Hospital, Changsha, China; 4 Xiangya School of Public Health, Central South University, Changsha, China; 5 Department of Epidemiology and Health Statistics, Medical College of Soochow University, Suzhou, China; 6 Hunan Provincial Key Laboratory of Pediatric Orthopedics, Hunan children’s Hospital, Changsha, China

**Keywords:** blood biochemical indicators, children, hypertension, obesity, restrictive cubic spline

## Abstract

**Introduction:**

Childhood hypertension and obesity are among the most prevalent health conditions worldwide, with obesity increasing the risk of hypertension in children. However, there has been limited focus on children with obesity.

**Methods:**

To identify the risk factors of hypertension in children with obesity and the relationship between them, we collected information from children who were registered at the Child Health Development Center of Hunan Children's Hospital between October 2021 and December 2023. Logistic regression combined with restricted cubic spline was used to examine the relationship between the variables and hypertension in children with obesity.

**Results:**

A total of 756 children with obesity were included in the study. The detection rate of hypertension in children with obesity was 20.6%, with a mean age of 11.9±2.0 years. The logistic regression indicated that neutrophils (NE), the ratio of neutrophils to lymphocytes (NLR), platelet count (PLT), albumin (ALB) and triglycerides (TG) were independent risk factors for hypertension in children with obesity. Restricted cubic spline curves showed NE, NLR, PLT, ALB and TG increasing linearly with the risk of hypertension. There was a significant linear relationship between NE, NLR, ALB, TG and systolic blood pressure (SBP)/diastolic blood pressure (DBP). A nonlinear dose-response relationship is observed between PLT and SBP, but no dose-response relationship between PLT and DBP.

**Discussion:**

Elevated levels of NE, NLR, PLT, ALB, and TG are associated with hypertension in children with obesity. Future studies need normal-weight controls to better examine these links.

## 1 Introduction

Childhood hypertension and obesity are two major and growing global public health concerns. The World Health Organization estimated that 37 million children under the age of 5 were overweight in 2022, with nearly half residing in Asia (1). In China, 19% of children aged 0−6 years and 10.4% of those aged 6–17 years were overweight or obese in 2020 (2). Obesity in childhood and adolescence increases the risk of developing non-communicable diseases such as type 2 diabetes and cardiovascular disease (1). Hypertension is a major contributor to the burden of cardiovascular disease in China (3), affecting 3.11% of Chinese children aged 6–18 years, equivalent to 6.8 million children in 2020 (4). Childhood hypertension not only harms vital organs but often persists into adulthood (5, 6). Studies have shown that primary hypertension in children is strongly linked to obesity (7, 8), with the risk of hypertension increasing as the degree of obesity rises (from normal weight to overweight, obese, and extremely obese) (8). Despite existing research on childhood hypertension, there has been limited focus on children with obesity, who are at a higher risk of developing hypertension. Abnormal lipid metabolism and blood counts have been associated with increased hypertension risk in this group (9), underscoring the importance of monitoring blood biochemistry in children with obesity (8).

This study aims to explore the risk factors for hypertension in children with obesity, with a particular focus on blood biochemical markers. We hope this research will inform early screening, prevention, and intervention strategies for childhood hypertension.

## 2 Methods

### 2.1 Study population

Children were identified from the electronic health records of the Health Management Center at Hunan Children's Hospital. All individuals who attended the center for routine health examinations between 1 October 2021, and 31 December 2023, were screened for eligibility.

**Figure 1:**
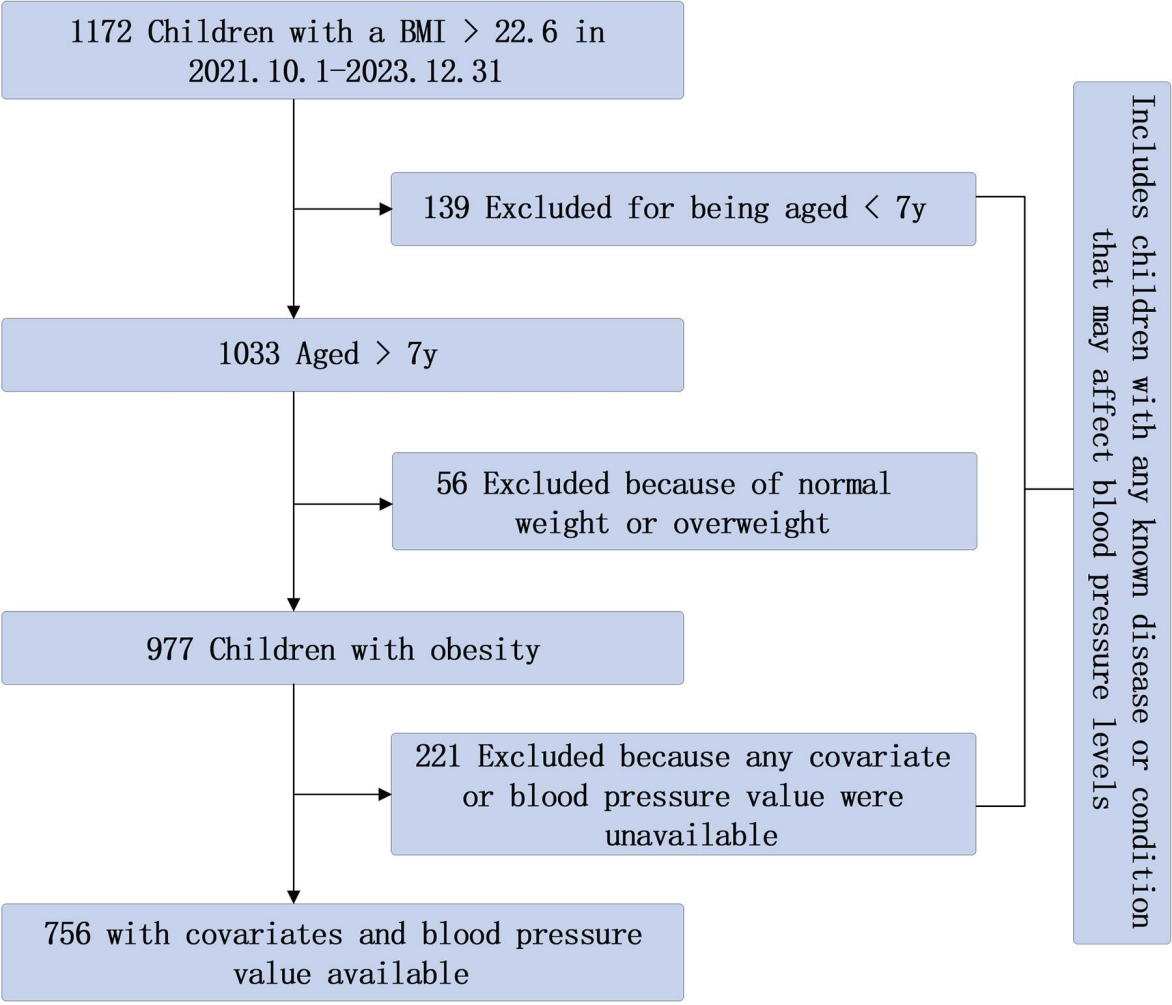
Flowchart of the study population, showing how the study subjects were selected.

The inclusion criteria were as follows: (i) registered for routine health check-ups at the Health Management Center; (ii) body mass index (BMI) > 22.6 kg/m^2^; and (iii) age > 7 years. The exclusion criteria were as follows: (i) presence of any known disease or condition that may affect blood pressure (BP) levels (e.g., renal disease, endocrine disorders, cardiovascular disease, or current use of antihypertensive medication); (ii) missing data for any covariate or blood pressure value; and (iii) children classified as normal weight or overweight, including those with a BMI greater than 22.6 who did not meet the obesity definition applied in this study (Figure 1).

Ethical approval for this study was granted by the Medical Ethics Committee of Hunan Children's Hospital (KYSQ2024-77), and informed consent was obtained from the parents of all participating children.

### 2.2 Data collection

We gathered clinical and demographic variables such as (i) age at the time of assessment; (ii) gender; (iii) height, weight, and BMI; (iv) BP; (v) blood biochemical indicators (e.g., liver and kidney function, blood sugar, and plasma lipid); and (vi) blood cell counts.

Height was measured using a stadiometer (Wuxi Hengqi Factory Co., Ltd., Wuxi, China) with participants standing barefoot and without hats, recorded to the nearest 0.1 cm. Weight was measured with children in light clothing, standing barefoot on a scale (Wuxi Hengqi Factory Co., Ltd., Wuxi, China), recorded to the nearest 0.1 kg.

BMI was then categorized according to cutoff values for “age- and sex-specific screening threshold for overweight and obesity of school-age children aged 6–18 years” as defined in *Screening for overweight and obesity among school-age children and adolescents* (10).

BP was measured by a single trained physician using an automated BP machine at the medical center. Children were required to rest quietly for at least 5 min before sitting, with their backs supported, feet together on the floor, right arm supported on a table at heart level, and left arm hanging relaxed by the side (the right arm was primarily used for measurements unless otherwise specified). A calibrated upper-arm electronic sphygmomanometer and an appropriate cuff size for children were used, with the lower edge of the cuff placed 1−2 cm above the elbow crease, allowing space for two fingers between the cuff and arm. The place of measurement was quiet (to eliminate the acute effect of noise) and comfortable (to eliminate the effects of cold or heat). Three measurements were obtained at 2-min intervals, and the average SBP and DBP were calculated.

According to the “age-, height- and sex-specific blood pressure reference criteria of children aged 3-17 years in China” in the the 2018 *Chinese guidelines for the management of hypertension* (11), hypertension is defined as a systolic blood pressure (SBP) and/or diastolic blood pressure (DBP) higher than the 95th percentile threshold corresponding to the subject's sex, age, and height.

### 2.3 Statistical analysis

The basic characteristics of the population (e.g., age, height, weight) were summarized using descriptive statistics. Categorical variables are presented as percentages, while continuous variables are expressed as the mean ± standard deviation (SD). Differences between children with normotension and those with hypertension were compared using independent-samples *t*-test for continuous variables and the *χ*^2^ tests for categorical variables. For blood biochemical indices, normally distributed variables were analyzed according to the same basic characteristics. Non-normally distributed data are reported as the median (interquartile range, IQR) [M (Q1, Q3)], and the differences between groups were assessed using the rank-sum test (Mann–Whitney *U*-test).

Hypertension risk factors were evaluated using binary logistic regression, with odds ratios (OR) and 95% confidence intervals (CIs) calculated. Restricted cubic splines were applied to assess potential non-linear relationships between independent risk factors and SBP, DBP, and hypertension risk, based on the logistic regression model. Descriptive and association analyses were performed using the IBM SPSS Statistics for Windows, version 26.0 (IBM Corp., Armonk, NY, USA). Restrictive cubic spline analysis was conducted using R software (version 4.4.1; R Foundation for Statistical Computing, Vienna, Austria) with the “rcssci” package. Differences with *P-*values of less than 0.05 were considered statistically significant.

## 3 Results

### 3.1 Differences between children with normotension and children with hypertension

A total of 1,172 children were enrolled in this study, of whom 756 children with obesity were included in the analysis. The mean age was 11.3 ± 1.82 years (range: 8–15 years), with girls representing 22.1% (*n* = 167). Among these children, 20.6% (*n* = 156) were diagnosed with hypertension. The mean SBP in children with hypertension was 128.0 ± 9.0 mmHg and the mean DBP was 73.7 ± 7.5 mmHg, which were 18 mmHg (*P* < 0.001) and 8.98 mmHg (*P* < 0.001) higher, respectively, than those of children with normotension.

In children with hypertension, age (11.9 vs. 11.2), proportion of children aged 12–15 years (59.6% vs. 40.4%), height (156.6 cm vs. 151.3 cm), weight (71.7 kg vs. 60.4 kg), BMI (28.8 vs. 26.1), SBP levels (128.0 mmHg vs. 110.0 mmHg), and DBP levels (73.7 mmHg vs. 64.8 mmHg) were significantly higher than in those without hypertension (*P* < 0.001); however, no significant differences were observed between sexes within either group (*P* = 0.739) (Table 1).

**Table 1:** Comparison of basic characteristics between hypertensive and normotensive groups of children with obesity.

Basic characteristics	All (*n* = 756)	Hypertension (*n* = 156)	Normotensive (*n* = 600)	*t/χ^2^/z*	*P*
Age	11.3 ± 1.82	11.9 ± 2.0	11.2 ± 1.7	4.248	**<0.001**
Age groups, *n* (%)				12.644	**<0.001**
8–11	401 (53.0)	63 (40.4)	338 (56.3)	——	——
>12	355 (47.0)	93 (59.6)	262 (43.7)	——	——
Sex, *n* (%)				0.111	0.739
Male	589 (77.9)	120 (20.4)	469 (79.6)	——	——
Female	167 (22.1)	36 (21.6)	131 (78.4)	——	——
Height	152.4 ± 11.1	156.6 ± 11.4	151.3 ± 10.7	5.456	**<0.001**
Weight	62.7 ± 15.0	71.7 ± 18.5	60.4 ± 13.0	7.196	**<0.001**
BMI	26.7 ± 3.3	28.8 ± 4.4	26.1 ± 2.7	7.317	**<0.001**
SBP	113.7 ± 10.9	128.0 ± 9.0	109.9 ± 7.9	24.709	**<0.001**
DBP	66.6 ± 7.3	73.7 ± 7.5	64.8 ± 6.0	15.708	**<0.001**

Data are presented as means ± SDs, medians (interquartile ranges), or *n* (%). Statistical comparisons were conducted only between the hypertensive and normotensive groups (independent-samples *t*-test, χ^2^ test or rank-sum test), not across three groups. Units of measurement: age in years; height in cm; weight in kg; BMI in kg/m^2^; SBP and DBP in mmHg. Age groups are presented in years (e.g., 8–11 means 8 to 11 years of age). For categorical variables (age groups, sex), data are shown as number of participants (*n*) with percentage (%) in parentheses. *n*, number of participants; %, percentage; BMI, body mass index; SBP, systolic blood pressure; DBP, diastolic blood pressure.

Bold values indicate variables with *P* < 0.05.

Compared with children with normotension, those with hypertension had significantly higher levels of NE (0.56 vs. 0.54), platelet count (PLT) (323.00 × 10^9^/L vs. 302.00 × 10^9^/L), and white blood cell count (WBC) (7.92 × 10^9^/L vs. 7.26 × 10^9^/L), while lymphocytes (LY) levels was lower (0.35 vs. 0.37), with statistically significant differences (*P* < 0.05) (Table 2).

**Table 2:** Comparison of blood cells between hypertensive and normotensive groups of children with obesity.

Blood cells	All (*n* = 756)	Hypertension (*n* = 156)	Normotensive (*n* = 600)	*z*	*P*
Neutrophils (NE)	0.54 (0.49, 0.60)	0.56 (0.50, 0.61)	0.54 (0.49, 0.59)	2.431	**0** **.** **015**
Lymphocytes (LY)	0.37 (0.31, 0.42)	0.35 (0.29, 0.40)	0.37 (0.32, 0.42)	−2.53	**0** **.** **011**
Platelet count (PLT)	305.00 (266.00, 344.00)	323.00 (267.00, 359.75)	302.00 (266.00, 340.00)	2.615	**0** **.** **009**
White blood cell count (WBC)	7.40 (6.37, 8.57)	7.92 (6.87, 9.25)	7.26 (6.28, 8.40)	4.438	**<** **0.001**
Serum hemoglobin (Hb)	136.0 (130.0, 142.0)	138.5 (132.3, 144.8)	135.0 (130.0, 141.0)	3.755	**<** **0.001**

Data are presented as medians (interquartile ranges). Statistical comparisons were conducted only between the hypertensive and normotensive groups (rank-sum test), not across three groups. Units of measurement: Platelet count and white blood cell count are presented in   ×  10⁹/L. Serum hemoglobin is presented in g/L.

Bold values indicate variables with *P* < 0.05.

Among children with hypertension, levels of albumin (ALB) (45.2 g/L vs. 44.2 g/L), total protein (TP) (74.6 g/L vs. 73.0 g/L), globulin (GLB) (29.7 g/L vs. 29.0 g/L), serum hemoglobin (Hb) (138.5 g/L vs. 135.0 g/L), aspartate aminotransferase (AST) (24.0 U/L vs. 22.3 U/L), alanine aminotransferase (ALT) (34.4 U/L vs. 24.4 U/L), total bile acids (TBA) (3.1 μmol/L vs. 2.8 μmol/L), uric acid (UA) (399.0 μmol/L vs. 345.0 μmol/L), glucose (GLU) (4.7 mmol/L vs. 4.7 mmol/L), triglycerides (TG; 1.2 mmol/L vs. 1.0 mmol/L), and low-density lipoprotein (LDL-C) (2.5 mmol/L vs. 2.5 mmol/L) were significantly higher compared with those with normotension, whereas no significant differences were observed in the levels of total bilirubin, indirect bilirubin, GLB, urea nitrogen, creatinine, myoglobin, cholesterol, and high-density lipoprotein (Table 3).

**Table 3:** Comparison of biochemical indices between hypertensive and normotensive groups of children with obesity.

Biochemical indices	All (*n* = 756)	Hypertension (*n* = 156)	Normotensive (*n* = 600)	*t/z*	*P*
Total bilirubin	9.5 (7.6, 11.9)	9.9 (8.1, 12.6)	9.3 (7.5, 11.8)	1.695	0.090
Direct bilirubin	2.7 (2.1, 3.6)	2.8 (2.1, 3.7)	2.7 (2.0, 3.6)	1.289	0.198
Indirect bilirubin	6.7 (5.3, 8.5)	7.0 (5.6, 8.7)	6.6 (5.2, 8.5)	1.169	0.242
Albumin (ALB)	44.3 (42.8, 45.9)	45.2 (43.3, 46.6)	44.2 (42.6, 45.6)	3.759	**<0** **.** **001**
Total protein (TP)	73.2 (70.8, 76.4)	74.6 (71.6, 77.7)	73.0 (70.6, 76.1)	3.845	**<0** **.** **001**
Globulin (GLB)	29.1 ± 3.5	29.7 ± 3.7	29.0 ± 3.40	−2.499	**0**.**015**
Aspartate aminotransferase (AST)	22.6 (19.0, 28.5)	24.0 (20.3, 33.4)	22.3 (18.7, 27.6)	3.491	**<0** **.** **001**
Alanine aminotransferase (ALT)	25.9 (17.6, 42.7)	34.4 (22.3, 57.0)	24.4 (16.9, 38.2)	5.218	**<0** **.** **001**
Total bile acids (TBA)	2.9 (1.9, 4.3)	3.1 (2.0, 4.7)	2.8 (1.9, 4.1)	1.849	**0**.**064**
Urea nitrogen	4.4 (3.7, 5.1)	4.5 (3.7, 5.2)	4.4 (3.7, 5.1)	0.459	0.646
Creatinine	48.2 (43.5, 55.3)	50.0 (43.9, 57.4)	48.0 (43.5, 54.7)	1.799	0.072
Uric acid (UA)	351.0 (305.0, 424.5)	399.0 (327.5, 474.8)	345.0 (301.0, 412.0)	4.831	**<0** **.** **001**
Glucose (GLU)	4.7 (4.4, 4.9)	4.7 (4.5, 5.0)	4.7 (4.4, 4.9)	1.982	**0**.**047**
Triglycerides (TG)	1.1 (0.8, 1.5)	1.2 (0.9, 1.7)	1.0 (0.8, 1.5)	3.489	**<0** **.** **001**
Cholesterol	4.2 (3.7, 4.7)	4.2 (3.7, 4.8)	4.2 (3.7, 4.6)	1.000	0.317
High-density lipoprotein	1.2 (1.0, 1.3)	1.1 (1.0, 1.3)	1.2 (1.0, 1.3)	−0.417	0.677
Low-density lipoprotein (LDL-C)	2.5 (2.1, 3.0)	2.5 (2.1, 3.1)	2.5 (2.0, 2.9)	1.593	**0**.**111**

Data are presented as means ± SDs or medians (interquartile ranges). Statistical comparisons were conducted only between the hypertensive and normotensive groups (independent-samples *t*-test or rank-sum test), not across three groups. Total bilirubin, direct bilirubin, indirect bilirubin, total bile acids, creatinine, and uric acid are presented in μmol/L. Albumin, total protein, and globulin are presented in g/L. Aspartate aminotransferase and alanine aminotransferase are presented in U/L. Urea nitrogen, glucose, triglycerides, cholesterol, high-density lipoprotein, and low-density lipoprotein are presented in mmol/L.

Bold values indicate variables with *P* < 0.05.

### 3.2 Analysis of risk factors for hypertension in children with obesity

Univariate logistic regression analysis indicated that older age, greater height, weight, and BMI, as well as elevated levels of NE, NLR, Hb, PLT, ALB, TP, GLB, AST, ALT, UA, GLU, and TG, were associated with an increased risk of hypertension in children with obesity (*P* < 0.05), whereas higher LY levels were associated with a decreased risk (*P* < 0.05) (Table 4).

**Table 4:** Univariate and multivariate logistic regression analysis of hypertension in children with obesity.

Variant	Univariate logistic regression		Multivariate logistic regression	
	OR (95% CI)	*P*	OR (95% CI)	*P*
Female	1.074 (0.706–1.634)	0.739	——	——
Age	1.245 (1.130–1.371)	**<0.001**	0.904 (0.760–1.076)	0.257
Height	1.045 (1.028–1.063)	**<0.001**	1.003 (0.877–1.148)	0.963
Weight	1.049 (1.036–1.061)	**<0.001**	1.021 (0.870–1.197)	0.800
BMI	1.256 (1.188–1.327)	**<0.001**	1.181 (0.788–1.769)	0.420
Neutrophils	21.437 (2.192–209.685)	**0**.**008**	0.000055 (9.9272E−9–0.3099)	**0**.**026**
Lymphocytes	0.036 (0.003–0.396)	**0**.**006**	0.013 (0.000002–105.892)	0.343
NLR	1.459 (1.148–1.855)	**0**.**002**	2.495 (1.158–5.377)	**0**.**020**
Serum hemoglobin	1.040 (1.022–1.060)	**<0.001**	1.014 (0.991–1.038)	0.220
Platelet count	1.004 (1.001–1.007)	**0**.**006**	1.005 (1.002–1.008)	**0**.**002**
White blood cell count	1.023 (0.990–1.058)	0.170	——	——
Total bilirubin	1.030 (0.986–1.075)	0.181	——	——
Direct bilirubin	1.106 (0.981–1.248)	0.099	——	——
Indirect bilirubin	1.029 (0.972–1.090)	0.321	——	——
Albumin	1.145 (1.068–1.228)	**<0.001**	1.117 (1.013–1.232)	**0**.**027**
Total protein	1.089 (1.045–1.135)	**<0.001**	1.004 (0.946–1.065)	0.907
Globulin	1.066 (1.012–1.122)	**0**.**015**		
Aspartate aminotransferase	1.013 (1.004–1.022)	**0**.**004**	1.008 (0.984–1.032)	0.529
Alanine aminotransferase	1.007 (1.003–1.011)	**<0.001**	0.998 (0.988–1.009)	0.757
Total bile acids	1.042 (0.993–1.094)	0.092	——	——
Urea nitrogen	1.021 (0.860–1.211)	0.815	——	——
Creatinine	1.017 (1.000–1.035)	0.055	——	——
Uric acid	1.005 (1.003–1.006)	**<0.001**	1.001 (0.998–1.003)	0.646
Myoglobin	1.009 (0.996–1.021)	0.162	——	——
Glucose	1.540 (1.015–2.338)	**0**.**043**	1.539 (0.946–2.501)	0.082
Triglycerides	1.628 (1.283–2.067)	**<0.001**	1.421 (1.068–1.892)	**0**.**016**
Cholesterol	1.145 (0.917–1.429)	0.231	——	——
High-density lipoprotein	0.809 (0.371–1.761)	0.593	——	——
Low-density lipoprotein	1.233 (0.962–1.582)	0.098	——	——

BMI, body mass index; NLR, the ratio of neutrophils to lymphocytes.

Bold values indicate variables with *P* < 0.05.

The results from the multivariate logistic regression revealed that NE (OR = 0.000055, 95% CI: 9.9272E −0.3099), NLR (OR = 2.495, 95% CI: 1.158−5.377), PLT (OR = 1.005, 95% CI: 1.002−1.008), ALB (OR = 1.117, 95% CI: 1.013−1.232), and TG (OR = 1.421, 95% CI: 1.068−1.892) were independent risk factors for hypertension in children with obesity (Table 4).

### 3.3 Dose–Response relationships between risk factors and SBP/DBP/hypertension risk in children with obesity

**Figure 2:**
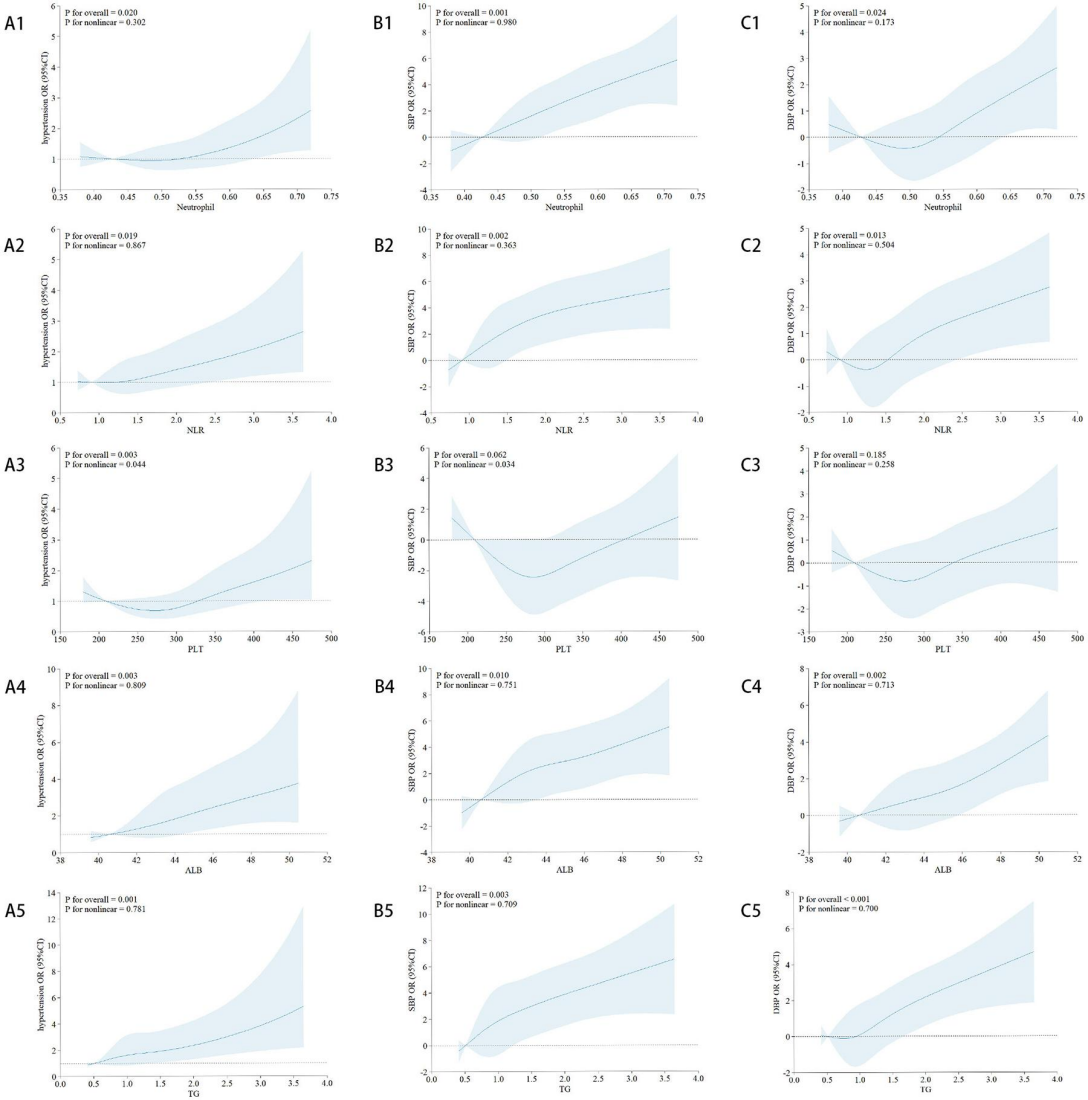
Relationship between neutrophils/NLR/PLT/ALB/TG and hypertension risk/SBP/DBP in children with obesity modeled using restricted cubic splines. The solid line represents the estimated effect, and the shaded area indicates the 95% confidence interval. *P* for overall tests the overall association, while *P* for non-linear tests whether the association deviates from linearity. **(A1−A5)** Correlation between hypertension risk and neutrophils/NLR/PLT/ALB/TG. **(B1−B5)** Correlation between SBP and neutrophils/NLR/PLT/ALB/TG. **(C1−C5)** Correlation between DBP and neutrophils/NLR/PLT/ALB/TG. NLR, the ratio of neutrophils to lymphocytes; PLT, platelet count; ALB, albumin; TG, triglycerides.

Based on multivariate logistic regression analysis, restricted cubic spline curves were constructed to visualize the dose–response associations. The results revealed linear relationships of NE, NLR, PLT, ALB, and TG with the hypertension risk (*P* overall < 0.05, *P* nonlinear > 0.05) (Figures 2A1–A5). SBP exhibited a linear increase in conjunction with rising levels of NE, NLR, ALB, and TG (*P* overall < 0.05, *P* nonlinear > 0.05), whereas a non-linear dose–response association was observed for PLT (*P* overall = 0.062, *P* nonlinear = 0.034) (Figures 2B1–B5). The restricted cubic spline analysis further indicated significant linear associations between NE, NLR, ALB, TG, and DBP (*P* overall < 0.05, *P* nonlinear > 0.05); however, no dose–response relationship was detected between PLT and DBP (Figures 2C1–C5).

## 4 Discussion

This study, conducted among children with obesity who underwent examination at the Health Management Center of Hunan Children's Hospital between October 2021 and December 2023, yielded several notable findings. First, 20.6% (*n* = 156) of children with obesity were diagnosed with primary hypertension; this group exhibited higher age, height, weight, and BMI compared with normotensive children. The blood biochemical indices NE, PLT, NLR, ALB, and TG were found to be associated with hypertension in children with obesity. A linear relationship was observed between NE, NLR, ALB, TG, and SBP/DBP, whereas a non-linear dose–response association was identified between PLT and SBP.

The results of this study suggest that NLR may be associated with the risk of hypertension in children with obesity and exhibits a linear relationship with both SBP and DBP. NE and lymphocytes are pivotal components of the immune system. NLR serves as a key hematological marker that reflects systemic inflammation and immune status (12). A meta-analysis of 13 pediatric studies reported that, in five studies assessing NE, the pooled mean NE level was significantly higher in the hypertensive group than in controls. Meanwhile, in six studies that evaluated NLR, the pooled results indicated a significantly increased mean NLR value in hypertensive children compared with the control group (13). NE may contribute to the development of arterial hypertension by promoting the release of reactive oxygen species and reactive nitrogen species, thereby exacerbating endothelial dysfunction. Under hypertensive conditions, NE can induce tissue inflammation and fibrosis (14).

In our analyses, PLT emerged as an independent risk factor for hypertension. This finding is consistent with that reported by Dziedzic-Jankowska et al. (13), who analyzed PLT across six pediatric studies involving a total of 615 participants. Their meta-analysis demonstrated that the pooled mean platelet count was significantly elevated in the primary hypertension group. Furthermore, previous studies have shown that elevated PLT is associated with various cardiovascular diseases. An increased PLT may exacerbate the inflammatory state, contributing to the progression of cardiovascular damage and the worsening of vascular lesions and injuries (15–18). Hypertension is a well-established major risk factor for cardiovascular disease. Collectively, these findings may provide insights into the relationship between PLT and hypertension in children with obesity.

A population-based cross-sectional study (the Norwegian Oslo Health Study) (19) demonstrated that ALB was positively associated with blood pressure across different age groups, and this association was independent of sex and age. This finding accords with our current observations, which showed that ALB was positively associated with the risk of hypertension in children with obesity. As ALB plays a crucial role in maintaining colloid osmotic pressure (20), this finding may be explained by a potential blood volume-expanding effect of ALB. However, as demonstrated by others, low ALB is considered a significant predictor in patients with cardiovascular disease (21), as ALB not only maintains osmotic pressure but also exerts anticoagulant and antiplatelet aggregation effects (20, 22, 23). This inconsistency may be attributed to the inherent limitations of cross-sectional study designs or to biological disparities between pediatric and adult populations. Accordingly, further well-designed investigations are warranted to elucidate the underlying mechanisms responsible for the elevated ALB levels observed in children with hypertension.

Another finding is that higher levels of TG were associated with increased risk of hypertension, and SBP/DBP increased linearly with higher TG levels in children with obesity. This finding is consistent with several previous studies reporting levels of TG and hypertension in children (24–26). Tong et al. (26) found that triglycerides ≥ 150 mg/dL (hypertriglyceridemia) were positively associated with hypertension and incorporated this parameter into a predictive model. Kim et al. (25), studying 1,858 children and adolescents aged 10–17 years who participated in the KNHANES 2016−2018, found that upward-reclassified youth showed elevated TG, higher BMI *z*-scores, and greater overweight/obesity compared with persistent normotensive youth [category created by 2016 European Society of Hypertension guidelines for the management of high blood pressure in children and adolescents (27)]. However, there was no difference in the levels of TG when upward-reclassified non-obese youth and persistent-normotensive non-obese youth were compared. Given our findings, we speculate that the relationship between TG and childhood hypertension may be influenced by obesity.

The association between obesity and hypertension has been well established. Previous studies have shown that obesity is frequently accompanied by low HDL-C levels and hypertriglyceridemia (28, 29). Elevated triglyceride levels may promote blood pressure elevation by inducing endothelial dysfunction, a process potentially mediated by small-dense LDL and/or fatty acids. In addition, an increase in circulating lymphocyte counts has been implicated as a potential mediator of triglyceride-associated hypertension (30). The connections among obesity, dyslipidemia, and hypertension are disturbing. Moreover, hypertriglyceridemia is commonly observed in children and adolescents with obesity and may elevate future cardiovascular risk (31), underscoring the importance of early detection, intervention, and preventive measures to reduce the risk of hypertension in children and adolescents with obesity and elevated TG levels.

Notably, chronic inflammation is strongly associated with hypertension in adults and contributes critically to its pathogenesis (32, 33). Accumulating evidence from *in vitro* experiments, rodent models, and human studies has demonstrated that inflammation and obesity are closely interrelated, with reciprocal interactions across multiple metabolic pathways (34). Given that NE, NLR, PLT, and ALB are inflammatory markers, it is possible that the association between these indicators and hypertension in children with obesity may be mediated through inflammatory mechanisms.

However, several limitations of this investigation must be noted. First, although this study is based on a specialized cohort, the data collected on this occasion were cross-sectional; therefore, we cannot define temporal associations for causal inference. Second, known confounding factors should be considered. Our study relied on documented medical examination data; thus, it was not possible to control for confounding factors such as family history of hypertension. Third, due to the single-center nature of our report, the sample may not be representative.

Moreover, a critical limitation of the present analysis is the exclusive inclusion of children with obesity and the consequent absence of a non-obese control group from the same setting. This precludes any direct comparison of blood pressure parameters or associated factors against a healthy reference baseline. Therefore, the observed statistical relationships pertain strictly to the internal heterogeneity of children with obesity and should not be extrapolated to the general population without a valid comparator. Nonetheless, this study is still a valuable exploration of the risk factors for hypertension in children with obesity in China.

## 5 Conclusion

NE, PLT, NLR, ALB, and TG were associated with the prevalence of hypertension, as well as SBP and DBP levels, in children with obesity. Owing to the cross-sectional design of this study, causality cannot be inferred. Future longitudinal cohort studies that include appropriate normal-weight comparators should further examine these relationships.

## Data Availability

The raw data supporting the conclusions of this article will be made available by the authors, without undue reservation.
